# Risk of hemorrhagic stroke in warfarin‐treated patients following heart valve replacement: Findings from the MAGPIE study

**DOI:** 10.1002/ibra.70020

**Published:** 2026-05-17

**Authors:** Dipannita Adhikary, Md. Abdullah Yusuf, AM Nayeem Parvez, Sheikh M. B. Faruque, Santo Barman, Adneen Moureen, Luke J. Rogers, Aziz Momin, Venkatachalam Chandrasekaran, Asit B. Adhikary, Redoy Ranjan

**Affiliations:** ^1^ Department of Biological Sciences Royal Holloway University of London London UK; ^2^ Department of Microbiology National Institute of Neurosciences and Hospital Dhaka Bangladesh; ^3^ Department of Cardiac Surgery Impulse Hospital and Research Centre Dhaka Bangladesh; ^4^ Department of Cardiac Surgery National Institute of Cardiovascular Diseases Dhaka Bangladesh; ^5^ Department of Microbiology Armed Forces Medical College Dhaka Bangladesh; ^6^ Department of Cardiothoracic Surgery, Academic Cardiovascular Unit South Tees NHS Foundation Trust Middlesbrough UK; ^7^ Department of Surgery Hull York Medical School York UK; ^8^ Department of Cardiac Surgery St George's University Hospitals NHS Foundation Trust London UK; ^9^ Department of Cardiac Surgery Bangabandhu Sheikh Mujib Medical University Dhaka Bangladesh

**Keywords:** Bangladesh, heart valve replacement, hemorrhagic stroke, mechanical valve, mortality

## Abstract

Hemorrhagic stroke (HS) remains a serious and potentially disabling complication following heart valve replacement (HVR). We aimed to evaluate the incidence and predictors of HS among mechanical HVR patients in Bangladesh. The Multidimensional Approach of Genotype and Phenotype In Stroke Etiology (MAGPIE) is an ambispective study recruited 568 consecutive HVR patients who had long‐term (≥6 months) warfarin therapy between January 2010 and December 2024. Among the 568 HVR patients, 4.4% experienced HS, with a mortality rate of 52%, and the median age was 40 years (interquartile range [IQR]: 32–45). The median duration of warfarin uses among mechanical heart valve patients with HS varied by procedure: 63 months (IQR: 48–86) for mitral valve replacement, 60 months (12–85) for aortic valve replacement, 96 months (52–101.50) for double valve replacement, and 42 months (24–60) for patients undergoing coronary artery bypass grafting (CABG) with valve replacement. Additionally, a receiver operating characteristic curve analysis identified 104 months as the discriminatory threshold for warfarin therapy duration in predicting post‐HVR HS onset. An age‐ and sex‐adjusted logistic regression model identified severe pulmonary hypertension (odds ratio [OR] 4.44; 95% confidence interval [CI] 1.15–17.04; *p* = 0.02) and warfarin therapy duration ≥104 months (OR 1.99; 95% CI 1.00–3.76; *p* = 0.04) as independent predictors of HS in patients with mechanical HVR. Severe pulmonary hypertension was associated with a 4.4‐fold higher risk and warfarin therapy beyond 104 months with a twofold higher risk of HS among patients with mechanical HVR.

## Graphical abstract

Among 568 mechanical heart valve replacement patients in Bangladesh, hemorrhagic stroke occurred in 4.4% with 52% mortality. Severe pulmonary hypertension increased risk 4.4‐fold, and warfarin therapy ≥104 months nearly doubled the risk, highlighting the need for long‐term risk monitoring in anticoagulated valve patients.

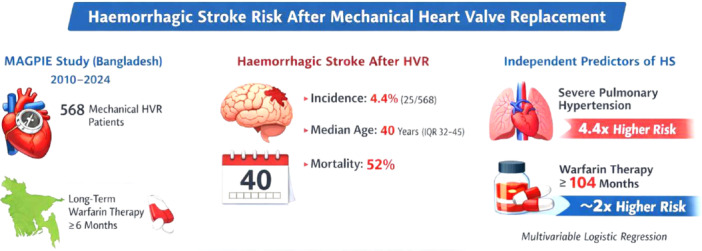

## 1 INTRODUCTION

Heart valve replacement (HVR) surgery has advanced considerably over recent decades, with both mechanical and biological prostheses offering distinct benefits and drawbacks. Mechanical valves, while highly durable, necessitate lifelong anticoagulation therapy because of their thrombogenic metallic components.^1^ In contrast, biological valves typically require anticoagulation for only the first three postoperative months during endothelialisation.^1^ Warfarin, a vitamin K antagonist, remains the standard anticoagulant for patients with mechanical valves, requiring regular International Normalised Ratio (INR) monitoring to maintain therapeutic levels between 2.0 and 3.5.^2^ Its management is challenging due to a narrow therapeutic window and multiple factors influencing dosage, including age, body weight, genetic polymorphisms, dietary vitamin K intake, and drug interactions.^3^, ^4^ In Bangladesh, genetic variations in VKORC1 significantly affect dose requirements, with approximately 80% of patients carrying the VKORC1 rs9923231 GG genotype requiring higher maintenance doses.^3^

The global burden of hemorrhagic stroke (HS) after valve replacement varies widely. International studies report annual hemorrhagic complication rates between 1.66% and 8.2% among warfarin‐treated patients.^5^, ^6^ A long‐term follow‐up study^5^ found hemorrhagic complications to be the most common adverse events, affecting 48.8% of patients with complications—significantly higher than thromboembolic events at 26.8%. Regional variation reflects differences in healthcare infrastructure, patient monitoring, and genetic influences on drug metabolism. In countries with established anticoagulation clinics, major bleeding occurs at 1.0% per patient‐year, compared with 4.9% in patients without specialised follow‐up.^7^ In developing regions, limited monitoring and patient education contribute to higher complication rates.^8^

Risk factors for HS in this context include age over 65 years, hypertension, and INR values 3.5 or more.^9^ Studies^8^, ^9^, ^10^ indicate that 75.8% of patients with systemic bleeding had INR levels above 2.5, while 72.7% had HAS‐BLED (Hypertension, Abnormal renal/liver function, Stroke, Bleeding history or predisposition, Labile INR, Elderly, Drugs/alcohol concomitantly) scores over 3. Mortality from anticoagulation‐related bleeding reaches about 10%.^11^ Poor anticoagulation control, defined as time in therapeutic range (TTR) below 45%, is associated with significantly higher risks of recurrent thromboembolism and major bleeding compared with TTR > 65%.^12^ Balancing thrombotic and bleeding risks is critical, as even temporary anticoagulation dysregulation can cause catastrophic thromboembolism or hemorrhage.^13^ Additionally, pulmonary hypertension may increase the risk of intracranial bleeding in anticoagulated mechanical valve patients through abnormalities in platelet aggregation and elevated right atrial and central venous pressures that impede cerebral venous drainage, raising intracranial venous pressure and making cerebral vessels more vulnerable to hemorrhage.^14^ Despite the substantial burden of valvular heart disease in South Asia, research on HS following valve replacement remains limited. This gap is notable given the predominance of rheumatic heart disease, younger patient age, differing genetic profiles, and distinct dietary habits compared with Western populations.^2^, ^15^, ^16^

This study evaluates the incidence, predictors, and impact of long‐term warfarin therapy on HS in Bangladeshi patients with mechanical heart valve replacement (m‐HVR). It identified population‐specific risk factors and assessed the effect of anticoagulation quality on bleeding outcomes, enhancing global understanding of stroke risk in diverse anticoagulated populations.

## 2 METHODS

### 2.1 Study design and data source

This ambispective cohort study was based on the Multidimensional Approach of Genotype and Phenotype In Stroke Etiology (MAGPIE) study, whose study protocol has been published elsewhere.^17^ A total of 568 patients who underwent HVR and received warfarin therapy for at least 6 months, with details of ≥6‐month follow‐up, from January 2010 to December 2024. Surgical procedures included mitral valve replacement, aortic valve replacement, double valve replacement, and combined coronary artery bypass grafting (CABG) with mechanical valve replacement, in which patients required long‐term warfarin therapy. Ethical approval was obtained from the Institutional Review Board of the National Institute of Neurosciences and Hospital (NINS&H), Dhaka, Bangladesh (IRB/NINS/2023/315), and all procedures were conducted in accordance with the Declaration of Helsinki. Informed consent was obtained from all participants who required long‐term warfarin therapy, or from their legal representatives when patients were unable to provide consent because of coma, mechanical ventilation, or death. All data were de‐identified to ensure participant confidentiality. The study combined retrospective review of medical records with prospective follow‐up of newly enrolled patients to evaluate the impact of long‐term warfarin therapy on HS in m‐HVR patients. This design enabled both historical trend assessment and real‐time outcome monitoring.

### 2.2 Study population

Inclusion criteria were age ≥18 years, mechanical valve replacement requiring long‐term warfarin therapy for at least 6 months with ≥6‐month follow‐up, and complete records including demographics, comorbidities, warfarin dosing, INR data, and neurological assessments. Exclusion criteria included congenital or acquired bleeding disorders, concurrent antiplatelet therapy, severe hepatic or renal dysfunction (estimated glomerular filtration rate [eGFR] <30 mL/min/1.73 m²), intracranial neoplasm or arteriovenous malformation, recent traumatic brain injury, participation in other trials, warfarin discontinuation within 30 days postsurgery, and loss to follow‐up. The primary outcome was to determine the incidence of HS onset among the m‐HVR population, confirmed through neurological evaluation and neuroimaging (computed tomography [CT] or magnetic resonance imaging [MRI]). Secondary outcomes included the incidence of ischemic stroke, major bleeding as defined by International Society on Thrombosis and Hemostasis (ISTH) criteria, all‐cause mortality, and identification of independent predictors of HS onset within the study cohort. We defined warfarin duration as the time from starting the medication after HVR until either all‐cause death or the onset of HS. Survival duration was measured from the date of surgery to either the date of death from any cause or the last follow‐up date, whichever came later. Pulmonary hypertension was evaluated by transthoracic echocardiography following the standard protocol, and severe pulmonary hypertension was defined as a pulmonary artery systolic pressure exceeding 70 mmHg.^18^ We calculated the mean values of INR, variability coefficients, and time in TTR using the Rosendaal method.^9^ The target INR levels for each type of prosthesis were set according to standard guidelines for aortic, mitral, double valve, or CABG with valve procedures.^4^, ^5^, ^6^, ^7^, ^8^, ^9^, ^11^

### 2.3 Statistical analysis

Analyses were conducted using SPSS *v*28.0 (IBM Corp.), with *p* < 0.05 considered statistically significant. Descriptive statistics were expressed as medians (interquartile ranges [IQR]) for continuous variables and frequencies (percentages) for categorical variables. Univariate analyses employed the Mann–Whitney *U* and chi‐square tests, with Fisher's exact test applied when the sample size was <5. Variables with *p* < 0.20 entered multivariate logistic regression to identify independent HS predictors, expressed as odds ratios (OR) with 95% confidence intervals (CI). Receiver operating characteristic (ROC) analysis determined the optimal warfarin duration cut‐off, with area under the curve (AUC) and sensitivity/specificity reported. Missing data were tested with Little's MCAR (missing completely at random) test, and multicollinearity was assessed using variance inflation factors (VIF > 4.0 indicating significance) as well as collinearity tolerance.

## 3 RESULTS

### 3.1 Baseline characteristics

**Figure 1:**
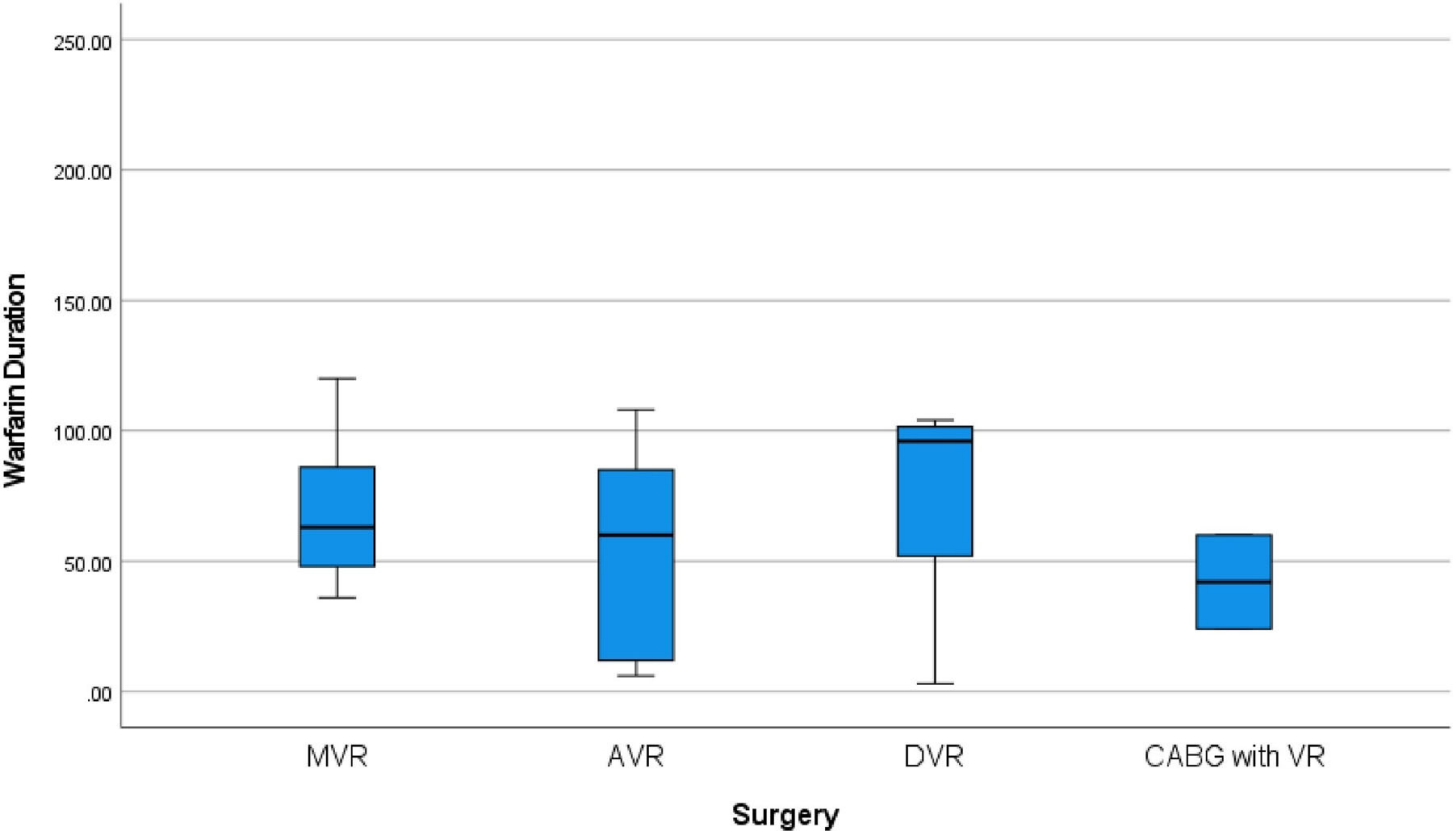
The box and whisker plot chart illustrate warfarin duration among hemorrhagic stroke samples in different surgical techniques. AVR, aortic valve replacement; CABG with VR, coronary artery bypass graft with valve replacement; DVR, double valve replacement; MVR, mitral valve replacement.

Among 568 m‐HVR patients, 25 (4.4%) experienced HS during follow‐up, with a median age of 40 years (IQR 32–45) (Table 1). The cohort's median age was 39 years (IQR 30–49), with a male predominance (62.7%). Mitral valve replacement was the most frequent surgical procedure (54.2%), followed by aortic valve (24.8%), double valve (15.0%), and combined CABG with valve replacement (5.8%). Overall, all‐cause mortality occurred in 83 cases (14.6%), while the prevalence of ischemic stroke was limited to four cases (0.7%). However, the mortality rate among m‐HVR with the HS population was 52%. Median survival for the cohort was 108 months (IQR 48–127), with no significant difference between those with and without HS (88 vs. 109 months; *p* = 0.60). Additionally, warfarin exposure among patients with HS varied by procedure: double valve replacement (96 months; IQR 52–101.5), mitral valve replacement (63 months; IQR 48–86), aortic valve replacement (60 months; IQR 12–85), and combined CABG with valve replacement (42 months; IQR 24–60) (Figure 1).

**Table 1:** Basic characteristics of study population (*n* = 568).

Variables		Total sample (*n* = 568)	Prosthesis with HS (*n* = 25)	Prosthesis without HS (*n* = 543)	*p* Value
Age (median [IQR]) years		39 (30–49)	40 (32–45)	40 (29‐48)	0.86
Age	MVR	36 (29.5–45)	39 (35–42)	39 (28–45)	0.75
	AVR	39.50 (30–50)	33 (30–39)	35.5 (27–42)	0.67
	DVR	40 (34–46)	42 (32.5–44)	41 (35.5–48)	0.62
	CABG + VR	57 (53–60)	67 (67–67)	57.5 (52.5–60)	0.04
Sex (men)		356 (62.7%)	20 (13.2%)	131 (86.8%)	0.052
Blood group	O group	66 (11.6%)	2 (8.7%)	21 (91.3%)	0.98
	non‐O group	143 (25.2%)	6 (8.6%)	64 (91.4%)	
Surgery	MVR	308 (54.2%)	10 (7.0%)	132 (93.0%)	0.21
	AVR	141 (24.8%)	6 (12.0%)	44 (88.0%)	
	DVR	85 (15.0%)	7 (16.3%)	36 (83.7%)	
	CABG + VR	33 (5.8%)	2 (20%)	8 (80%)	
Severe pulmonary HTN		30 (5.3%)	3 (23.1%)	10 (76.9%)	0.13
Hypertension		30 (5.3%)	6 (20%)	24 (80%)	0.09
Diabetes mellitus		24 (4.2%)	4 (16.7%)	20 (83.3%)	0.28
Warfarin		197 (34.7%)	20 (10.4%)	173 (89.6%)	0.87
Survival (median [IQR]) months		108 (48–127)	88 (54–119)	109 (47–127)	0.60

*Note*: The *p*‐values were calculated using the chi‐square test, Fisher's exact test, or the Mann–Whitney *U* test as appropriate, with values below 0.05 considered statistically significant.

Abbreviations: AVR, aortic valve replacement; CABG + VR, coronary artery bypass graft with valve replacement; DVR, double valve replacement; IQR, interquartile range; MVR, mitral valve replacement.

### 3.2 Optimal warfarin therapy cut‐off duration

**Figure 2:**
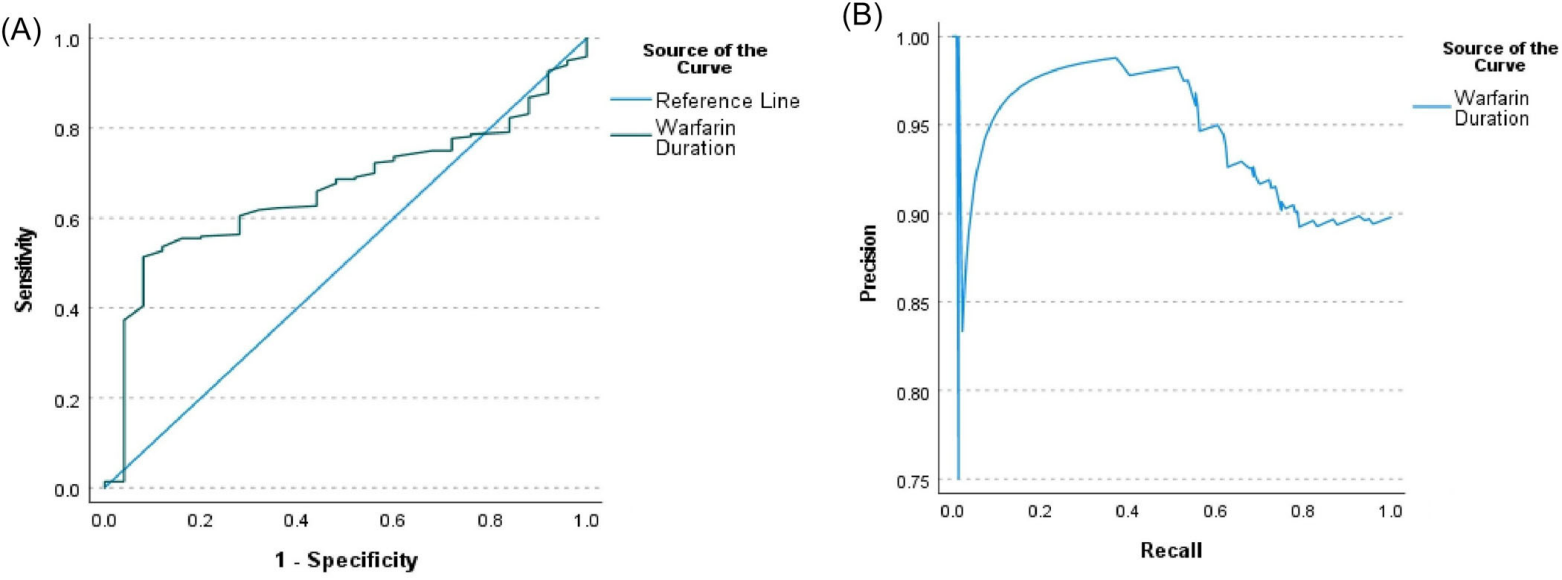
Receiver operating characteristic (ROC) curve (A) and precision–recall curve (B) for predicting HS following m‐HVR based on warfarin therapy duration. The ROC analysis identified an optimal cut‐off of 104 months. HS, hemorrhagic stroke; m‐HVR, mechanical heart valve replacement.

The ROC curve analysis indicated 104 months as the optimal warfarin therapy cut‐off for predicting HS following m‐HVR (AUC 0.663; 95% CI 0.57–0.75; *p* < 0.001), with 68.6% sensitivity and 48.0% specificity (Figure 2A). The positive predictive value was 98.3%, with a recall sensitivity of 51.4% (Figure 2B). Further, internal validation was performed using nonparametric bootstrapping to cross‐validate the stability of the optimal cut‐off points for warfarin duration and AUC. The bootstrapped analysis demonstrated a mean optimal cut‐off of 103.6 months (95% CI 96.2–111.4 months). The optimism‐corrected AUC was 0.658 (95% CI 0.572–0.742), indicating moderate discrimination. At this threshold, the mean sensitivity was 66.9% (95% CI 60.1%–73.5%) and the mean specificity 49.3% (95% CI 42.0%–56.8%). The positive predictive value remained high (97.9%, 95% CI 96.8%–98.8%), although recall sensitivity was modest. Overall, bootstrap validation suggests that the identified cut‐off is stable, with moderate variability consistent with the observed discriminatory performance.

### 3.3 Independent predictors of HS among m‐HVR population

Multivariate logistic regression, adjusted for demographic and clinical variables, identified severe pulmonary hypertension (OR 4.44; 95% CI 1.15–17.04; *p* = 0.02) and warfarin therapy duration ≥104 months (OR 1.99; 95% CI 1.0–3.76; *p* = 0.04) as the strong independent predictors of m‐HVR with HS (Table 2). Further, goodness‐of‐fit of the risk prediction model was good in predicting HS based on post‐m‐HVR warfarin therapy duration (Supporting Information S1: Figure S1). Little's MCAR test indicated that missing data were random (*χ*² = 14.32, *df* = 12, *p* = 0.281). Further, there was no evidence of significant multicollinearity among the covariates, as indicated by variance inflation factors (VIFs < 4.0) and acceptable collinearity tolerance values (>0.25) across all variables.

**Table 2:** An age and sex adjusted multivariate logistic regression model identified independent predictors of post‐HVR hemorrhagic stroke.

Variables	*p* Value	Odds ratio (OR)	95% Confidence interval	
			Lower	Upper
Age	0.66	1.02	0.94	1.09
Severe pulmonary hypertension	0.02	4.44	1.15	17.04
Warfarin duration ≥104 months	0.04	1.99	1.0	3.76
Hypertension	0.12	2.83	0.74	10.89
Diabetes mellitus	0.45	1.61	0.46	5.70

Abbreviation: HVR, heart valve replacement.

## 4 DISCUSSION

This study reports a 4.4% incidence of HS following mechanical HVR, identifies severe pulmonary hypertension as an independent predictor, and suggests a 104‐month warfarin threshold associated with a twofold increase in HS risk. These findings contribute valuable evidence to the limited literature on anticoagulation‐related complications in South Asian populations and should be interpreted within the unique clinical context of Bangladesh.

The findings of HS incidence in this study are consistent with international reports of bleeding events after valve replacement, though such rates vary widely between populations.^19^, ^20^, ^21^ Similarly, European studies found about 4% per patient‐year hemorrhagic event rate in artificial valve recipients.^22^, ^23^ A recent US prospective cohort study^24^ reported that 17% of patients aged 65 years or older who underwent aortic valve replacement for calcific aortic stenosis experienced clinical strokes, a rate substantially higher than that observed in the present study. This discrepancy may be attributable to the US study's focus on a specific population: patients aged 65 years or older undergoing aortic valve replacement for calcific aortic stenosis. Data from existing literatures suggests that proper warfarin administration and monitoring significantly reduce bleeding risk, highlighting the influence of monitoring quality.^25^, ^26^, ^27^ The Bangladeshi post‐HVR HS incidence likely reflects both the inherent risks of anticoagulation and limitations in monitoring infrastructure. Existing studies^28^, ^29^, ^30^ link bleeding risk primarily to INR control, patient comorbidities, and dosing factors, but the association with severe pulmonary hypertension with post‐HVR HS is novel. Despite severe pulmonary hypertension emerging as a strong predictor of HS, a relationship rarely addressed in previous research.^31^, ^32^ The haemodynamic alterations in severe pulmonary hypertension may influence coagulation parameters or vascular fragility, increasing bleeding risk through mechanisms not captured in standard risk models.^33^, ^34^ Additionally, pulmonary hypertension increases the risk of intracranial bleeding in anticoagulated mechanical valve patients by elevating right atrial and central venous pressures. This impedes cerebral venous drainage, raises intracranial venous pressure, and makes cerebral vessels more susceptible to hemorrhage during anticoagulation.^32^, ^33^, ^34^ Insofar as we know, the identification of severe pulmonary hypertension and the 104‐month warfarin cut‐off for post‐HVR HS has not been reported before in the literature.

The twofold increase in HS risk observed among patients with mechanical HVR after 104 months of warfarin therapy is primarily attributable to cumulative anticoagulant exposure and progressive deterioration in anticoagulation control.^12^, ^35^, ^36^ Greater INR variability and supratherapeutic episodes increase the risk of intracranial bleeding, while prolonged warfarin therapy further heightens the likelihood of adverse INR excursions.^3^, ^9^ A decrease in time within the therapeutic range is associated with a 34% higher hazard of major bleeding for every 10% reduction, and many hemorrhagic events occur at INR values of 3.2 or higher.^12^, ^13^, ^35^, ^36^, ^37^ Additionally, patient‐related factors that accumulate with age, such as declining renal function, vascular comorbidities, and concomitant antiplatelet or interacting medications, further increase bleeding susceptibility during extended anticoagulation.^26^, ^29^, ^38^ The cumulative effect of these risks explains why the median time to the first major bleeding event in long‐term cohorts extends to several years after valve implantation, with the absolute hemorrhage risk rising progressively with treatment duration.

This is the first comprehensive report of HS incidence with HVR in Bangladesh, filling an important gap in regional literature. The single‐centre, observational design introduces certain limitations, including potential selection bias and lack of randomisation, which restrict causal inference. Unmeasured factors—such as socioeconomic status, adherence, and healthcare access—may also have influenced outcomes. However, the single‐centre approach ensured consistent treatment protocols and follow‐up, a strength in the context of Bangladesh's variable healthcare delivery. Although patients were followed up with target INR monitoring and warfarin dose adjustment, the absence of actual INR values at the time of HS remains a limitation. Nevertheless, the primary objective was to assess the association between elevated INR (above the therapeutic target) and HS. As therapeutic ranges vary by valve position (e.g., aortic vs*.* mitral), INR was categorised as elevated or within the target range. Warfarin pharmacogenetic studies in Bangladesh have identified a high prevalence (~80%) of the VKORC1rs9923231 GG genotype, which may influence dosing and complication risk, similar to existing Asian publications.^3^, ^39^ The extended warfarin threshold identified here reinforces the need for prolonged monitoring and patient education beyond the 3–6 months emphasised locally in Bangladesh,^3^, ^14^, ^15^ and in international literature.^20^, ^21^, ^22^, ^23^, ^28^, ^29^ Establishing dedicated anticoagulation clinics, shown elsewhere to reduce HS rates from 4.9% to 1.0% per patient‐year, could improve outcomes.^26^, ^29^, ^40^ Further missing data might raise concerns regarding selective reporting bias; however, the nonsignificant Little's MCAR test mitigates this risk. Although the study is robust, it did not consider key modifiable factors directly linked to HS risk in prosthetic heart valve patients, including INR variability, interacting medications, organ dysfunction, and alcohol intake, which may have introduced residual confounding. Future prospective studies should include detailed anticoagulation quality metrics and clinical risk profiles to clarify causal associations and identify modifiable determinants of bleeding risk. Long‐term follow‐up systems and structured patient engagement are essential to mitigate risk and optimise management in resource‐limited settings.

## 5 CONCLUSION

HS occurred in 4.4% of Bangladeshi m‐HVR patients, with a 52% mortality rate. Severe pulmonary hypertension independently increased the risk 4.4‐fold, while warfarin therapy beyond 104 months was associated with a ~2‐fold higher risk of HS after m‐HVR. These findings emphasise the necessity for prolonged anticoagulation monitoring and population‐specific risk stratification, particularly considering genetic variations affecting warfarin metabolism in South Asian patients.

## AUTHOR CONTRIBUTIONS

Dipannita Adhikary, Md. Abdullah Yusuf, and Redoy Ranjan contributed to conceptualisation, recruitment, data analysis, picture production, and manuscript drafting. AM Nayeem Parvez, Sheikh M. B. Faruque, Santo Barman, Luke J. Rogers, and Adneen Moureen were responsible for data collection, methodology, and analysis. Aziz Momin, Venkatachalam Chandrasekaran, and Asit Baran Adhikary provided essential input, critical revisions, and supervision. All authors contributed to the article and approved the submitted version.

## CONFLICT OF INTEREST STATEMENT

The authors declare no conflicts of interest.

## ETHICS STATEMENT

Ethical approval was obtained from the Institutional Review Board of the National Institute of Neurosciences and Hospital (NINS&H), Dhaka, Bangladesh (IRB/NINS/2023/315), and all procedures were conducted in accordance with the Declaration of Helsinki. Informed consent was obtained from all participants or their legal representatives.

## Supporting information

Supplementary Figure S1.

## Data Availability

The data that support the findings of this study are available on request from the corresponding author. The data are not publicly available due to privacy or ethical restrictions.
