# Supplementary material for: Risk of hemorrhagic stroke in warfarin‐treated patients following heart valve replacement: Findings from the MAGPIE study
Source: Ibrain. 2026 May 17;12(2):250–8. doi: 10.1002/ibra.70020 (PMC13310240; doi:10.1002/ibra.70020)
Supplement: Supplementary file 1 — Supplementary Figure S1. [file IBRA-12-250-s001.docx]

**Figure S1:** The goodness-of-fit of the model in predicting haemorrhagic stroke based on post-HVR warfarin therapy duration.


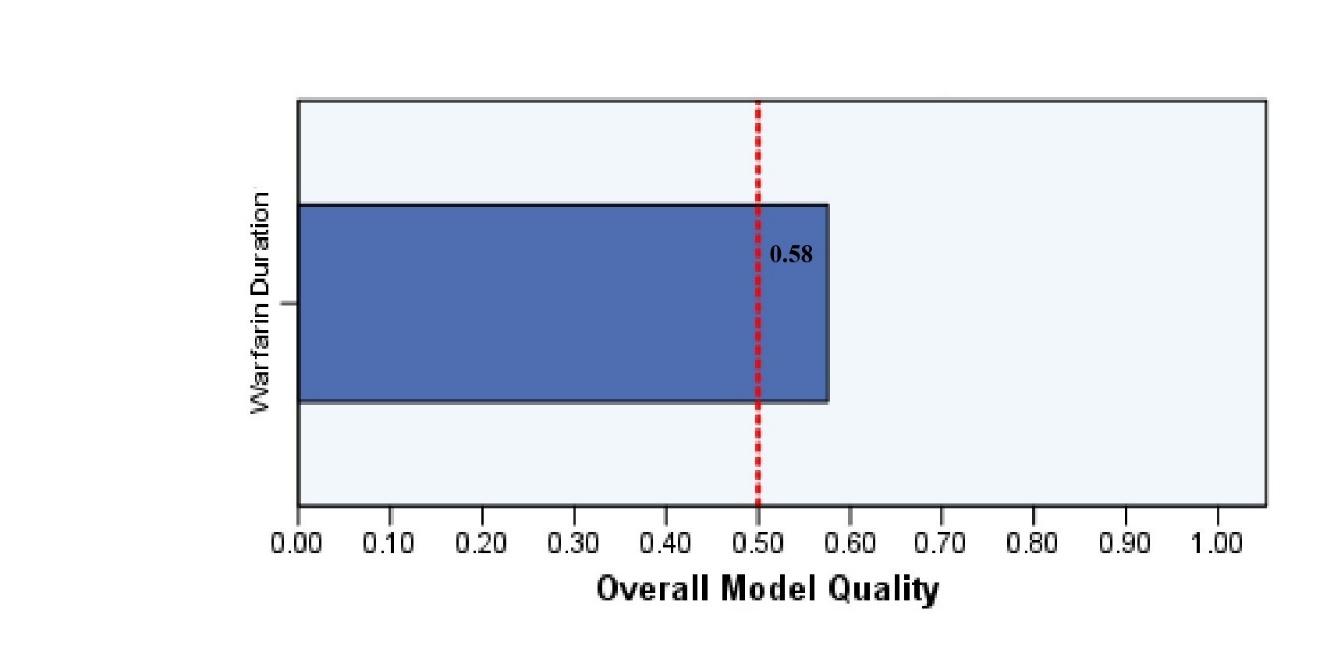


Note: The red dashed line indicates the model’s goodness-of-fit. A good model has a value >0.5, whereas a value <0.5 indicates the model is no better than a random prediction.
